# Supplementary material for: Oncogenic PI3Kα variants reveal graded conformational spectrum with mutation-specific cryptic pockets
Source: Commun Chem. 2026 Jan 21;9:100. doi: 10.1038/s42004-026-01906-x (PMC12923765; doi:10.1038/s42004-026-01906-x)
Supplement: Supplementary file 1 — Supplemental Material [file 42004_2026_1906_MOESM1_ESM.pdf]

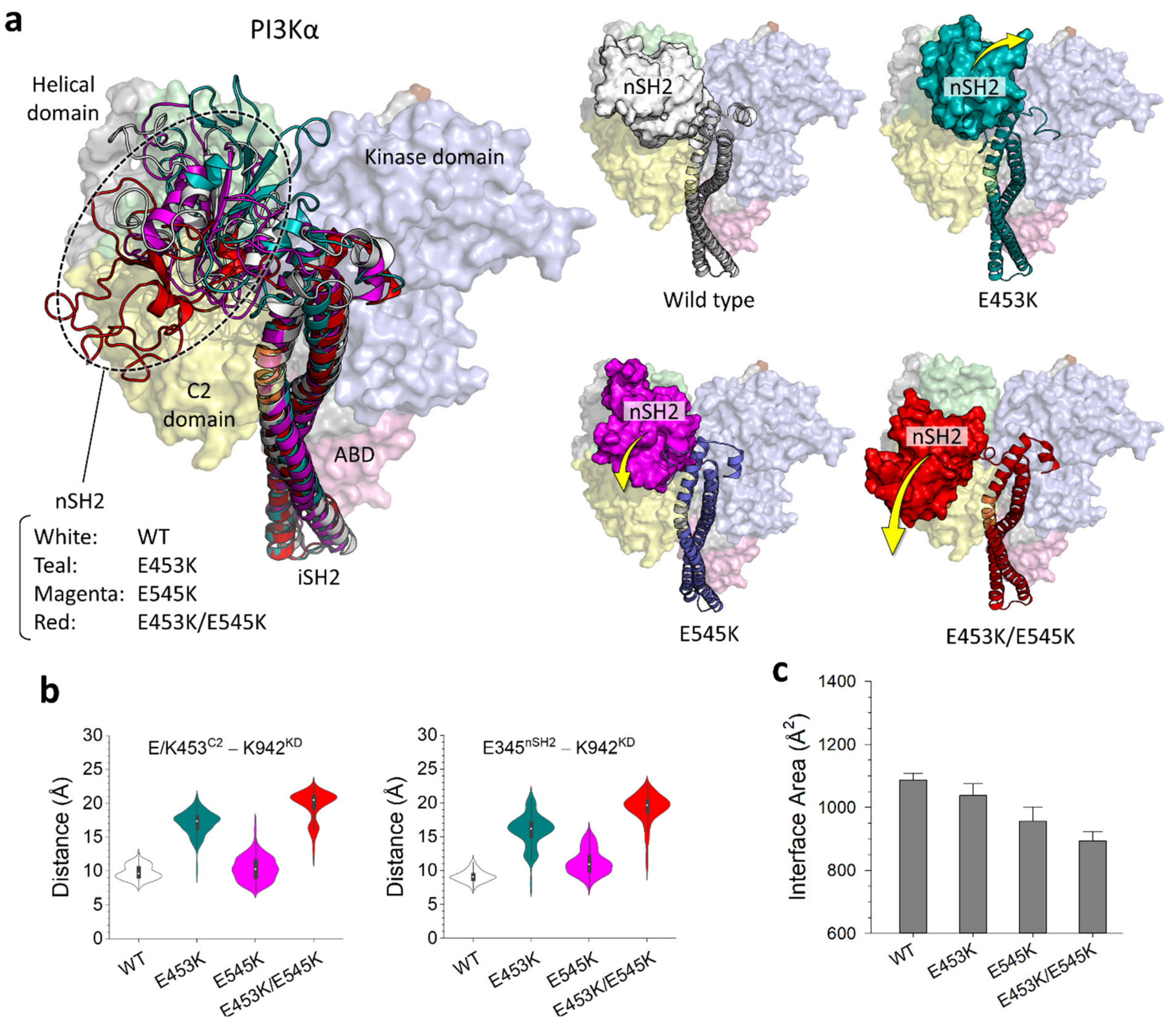

Supplementary Fig. 1 | The best representative conformations of PI3Kα variants compared with the wild type.

a, Superimposition of the best representative conformations of PI3Kα^{WT}, PI3Kα^{E453K}, PI3Kα^{E545K}, and PI3Kα$^{E453K/E545K}$ (*left*), along with their individual snapshots (*right*). Yellow arrows indicate the nSH2 movement relative to the wild type. The size of arrows refers to the strength of the movement. **b**, Violin plots representing the atomic pair distances of E/K453^{C2}–K942^{KD} and E345^{nSH2}–K942^{KD}. **c**, The surface area of the interface between nSH2 and the p110α subunit calculated using the PISA program (https://www.ebi.ac.uk/pdbe/pisa/).

.

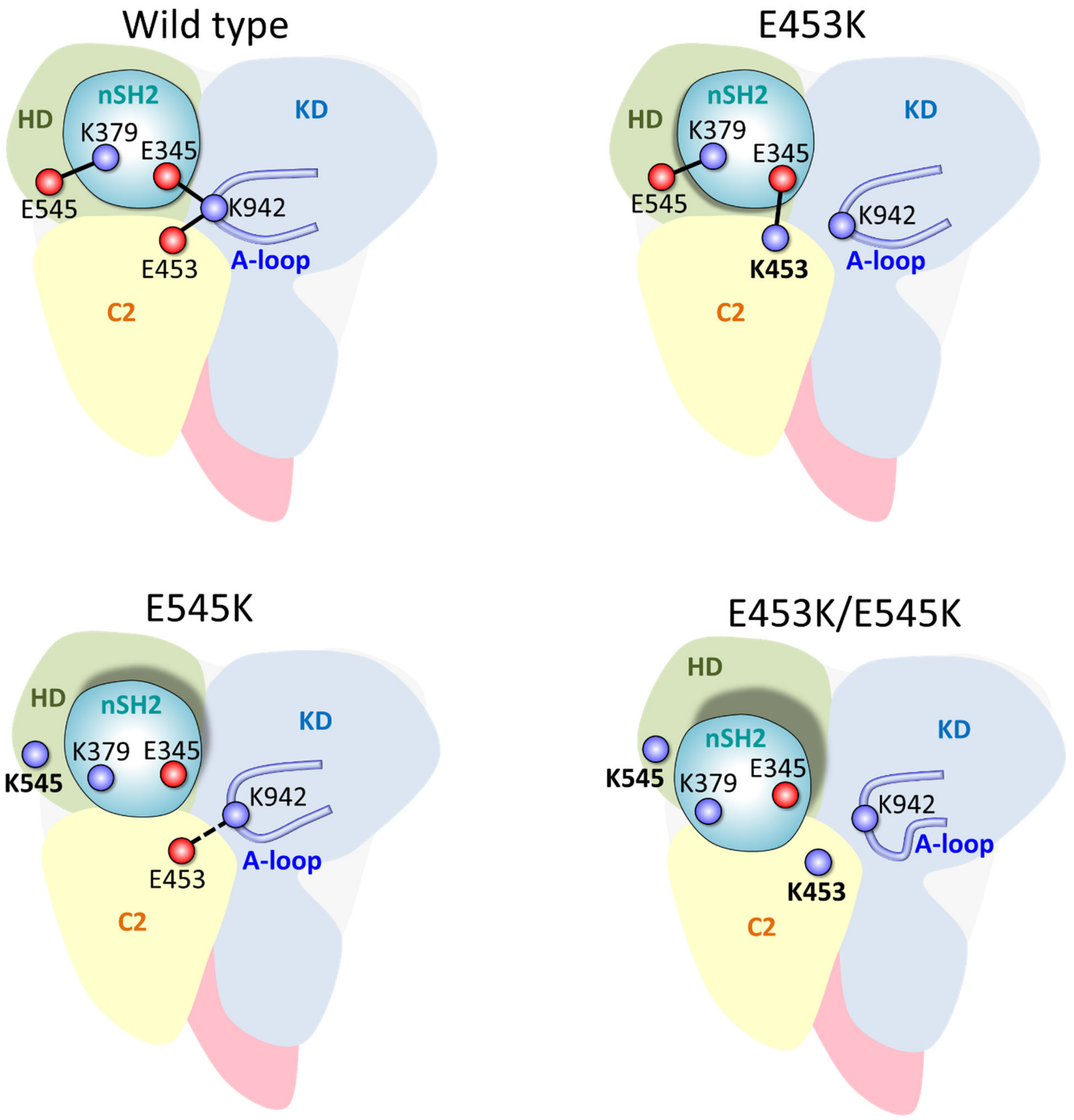

Supplementary Fig. 2 | The nSH2 release mechanism.
Schematic diagrams represent how the mutations in PI3Kα disrupt the salt bridge network at the nSH2/helical/C2/kinase domain interface. A significant conformational change in the A-loop can be observed upon nSH2 release.

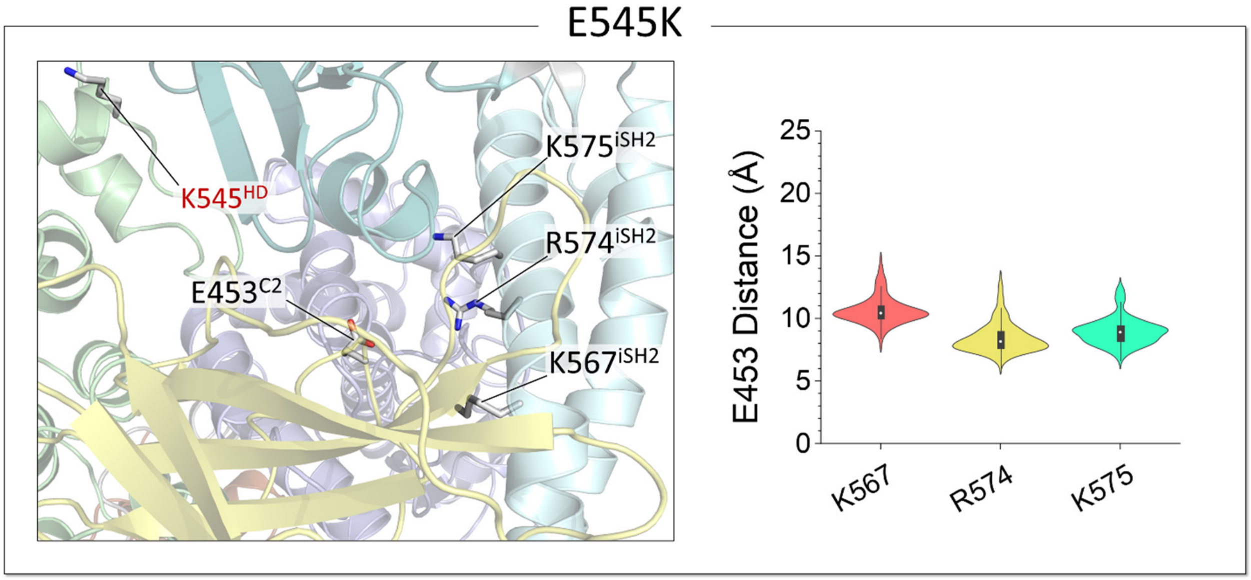

Supplementary Fig. 3 | The hotspot E545K mutation alone is less effective on the iSH2 movement.

Highlights of the salt bridge interactions at the iSH2/C2 domain interfaces for PI3Kα^{E545K} (*left panel*), and violin plots representing the atomic pair distances of E453^{C2}–K567^{iSH2}, E453^{C2}–R574^{iSH2}, and E453^{C2}– K575^{iSH2} (*right panel*). The protein structure is depicted as a cartoon with different colors: the C2 domain is yellow, the helix domain is green, the kinase domain is blue, and iSH2 is cyan.

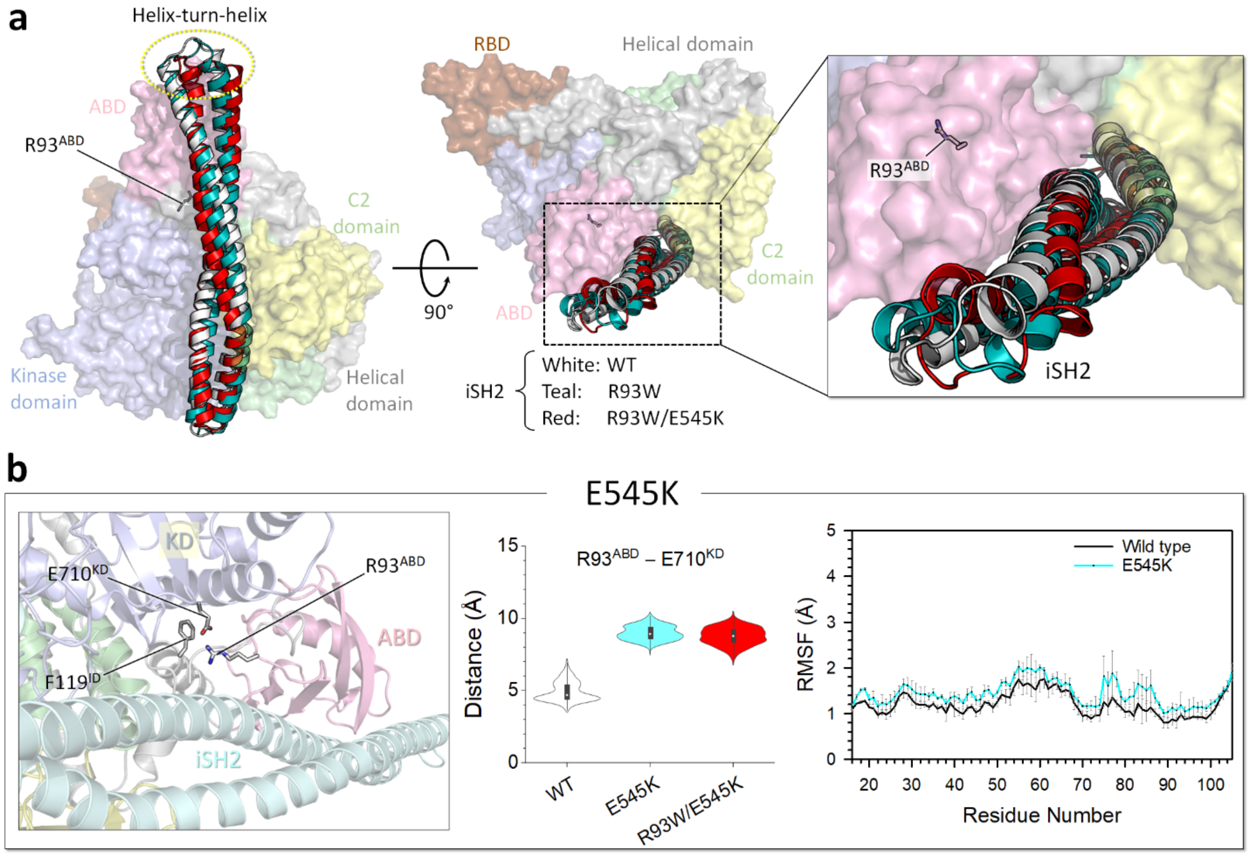

Supplementary Fig. 4 | The R93W mutation disrupts the ABD/kinase domain interface.
a, Superimposition of the best representative conformations of PI3Kα^{WT}, PI3Kα^{R93W}, and PI3Kα$^{R93W/E545K}$ highlighting the movement of the helix-turn-helix region of iSH2. The p110α is shown in surface representation with different color codes: The ABD is pink, the RBD is brown, the C2 domain is yellow, the helical domain is green, and the kinase domain is blue. The iSH2 structure is depicted as a cartoon, showing the wild type in white, the single mutation R93W in teal, and the double mutation R93W/E545K in red. **b**, The best representative conformation from the ensemble clusters for PI3Kα^{E545K} is shown, with highlights of the residue interactions at the ABD/kinase domain interfaces (*left*). Violin plot representing the atomic pair distance of R93^{ABD}–E710^{KD} compared with the wild type and the double mutation R93W/E545K (*middle*). The root-mean-squared-fluctuation (RMSF) of the ABD for PI3Kα^{E545K} (cyan) compared with the wild type (black) (*right*).

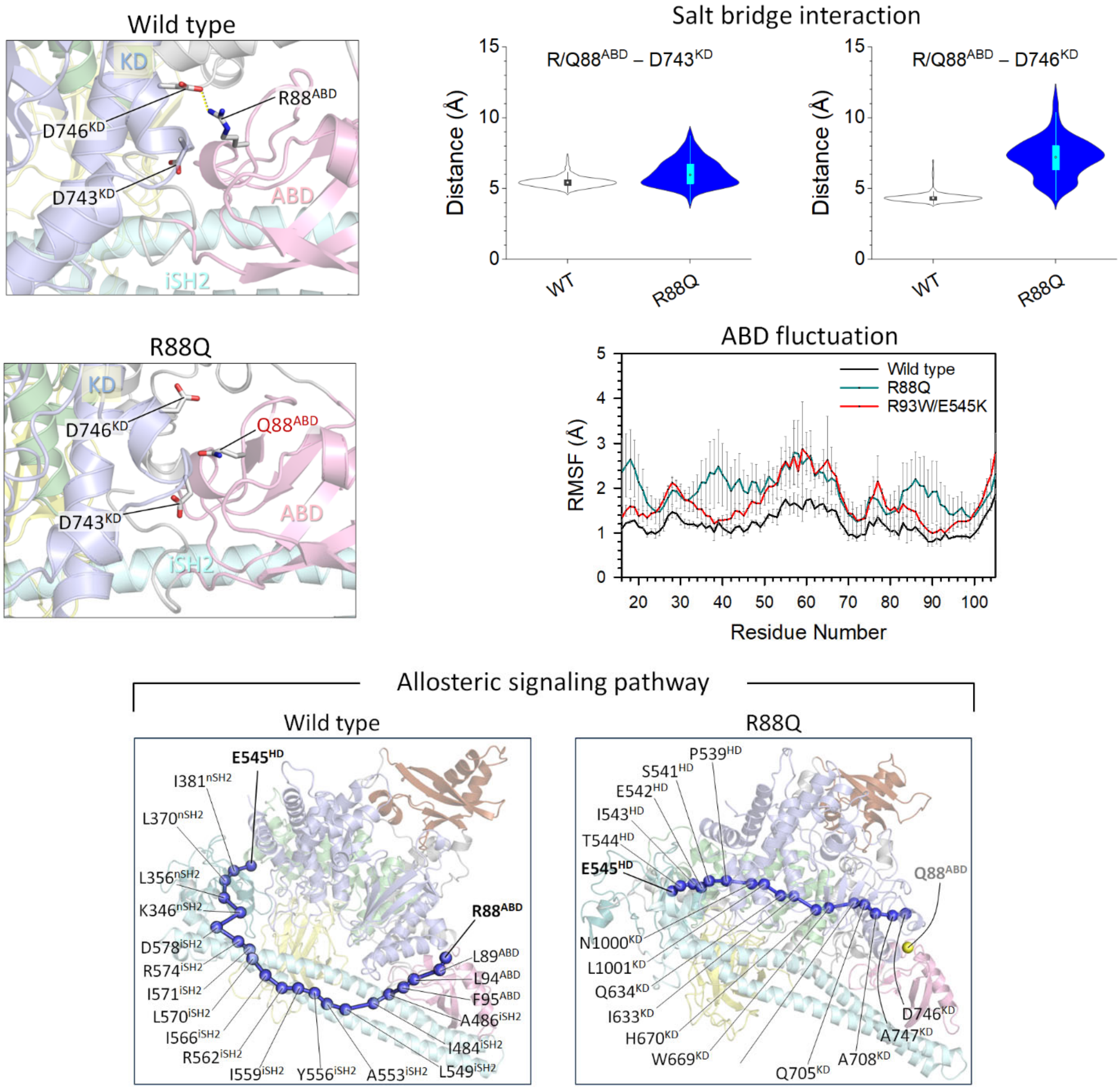

Supplementary Fig. 5 | The R88Q mutation also disrupts the ABD/kinase domain interface. The best representative conformations from the ensemble clusters for PI3Kα^{WT} and PI3Kα$^{\mathrm{R88Q}}$ are shown, with highlights of the residue interactions at the ABD/kinase domain interfaces (*left*). The protein structure is depicted as a cartoon with different colors: the ABD is pink, the helix domain is green, the kinase domain is blue, and iSH2 is cyan. Violin plots representing the atomic pair distances of R/Q88^{ABD}–D743^{KD} and R/Q88^{ABD}–D746^{KD} (*right*). The root-mean-squared-fluctuation (RMSF) of the ABD for PI3Kα$^{\mathrm{R88Q}}$ (teal) compared with that for PI3Kα^{WT} (black) and PI3Kα$^{\mathrm{R93W/E545K}}$ (red) (*middle*). The allosteric signaling pathways between the source residue E545^{HD} and the sink residue R/Q88^{ABD}. Blue beads denote the allosteric signal nodes, and yellow bead for Q88^{ABD} in the R88Q mutation indicates the broken allosteric signal.

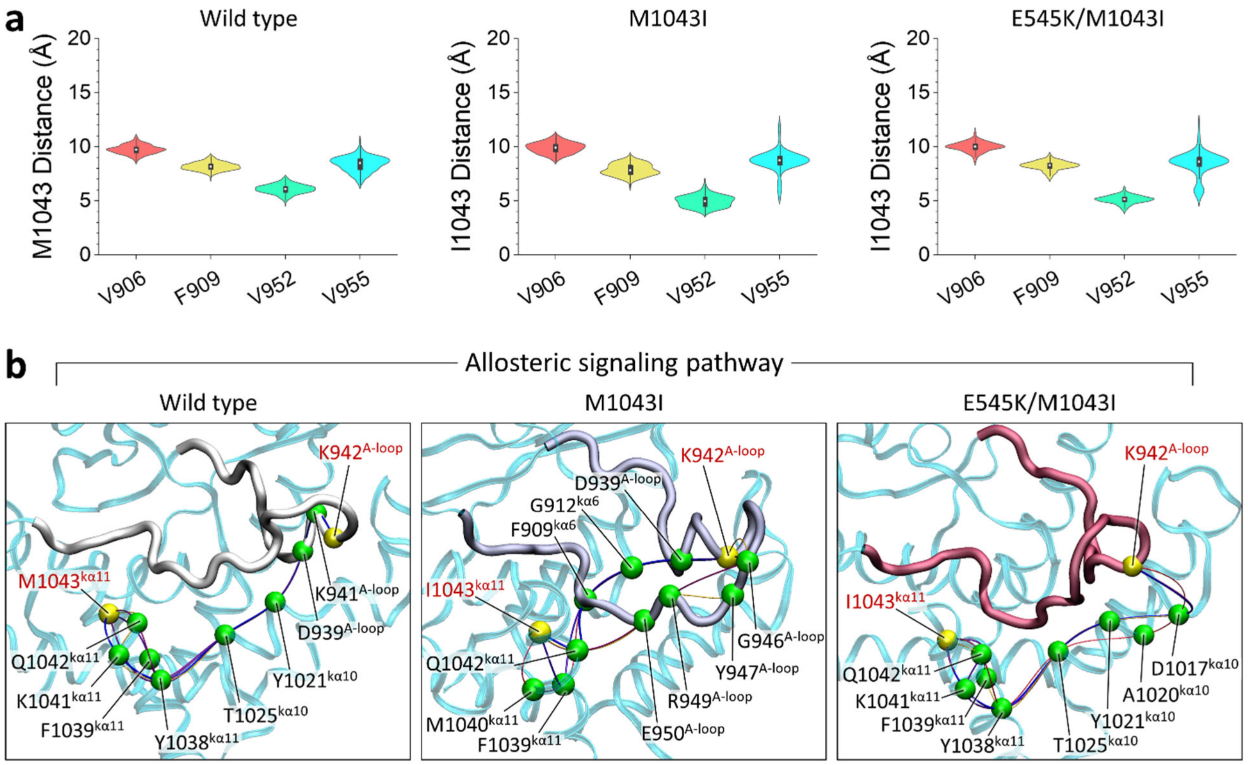

Supplementary Fig. 6 | The M1043I mutation affects the A-loop conformation.
a, Violin plots representing the atomic pair distances of M/I1043$^{k\alpha11}$–V906$^{k\alpha6}$, M/I1043$^{k\alpha11}$–F909$^{k\alpha6}$, M/I1043$^{k\alpha11}$–V952$^{A\text{-}loop}$, and M/I1043$^{k\alpha11}$–V955$^{A\text{-}loop}$ for the wild type, the single mutation M1043I, and the double mutation E545K/M1043I. Both mutations preserve the hydrophobic cluster, similar to the wild type. **b**, The allosteric signaling pathways between the source residue M/I1043$^{k\alpha11}$ and the sink residue K942$^{A\text{-}loop}$. Yellow beads represent the source and sink residues, while green beads denote the allosteric signal nodes.

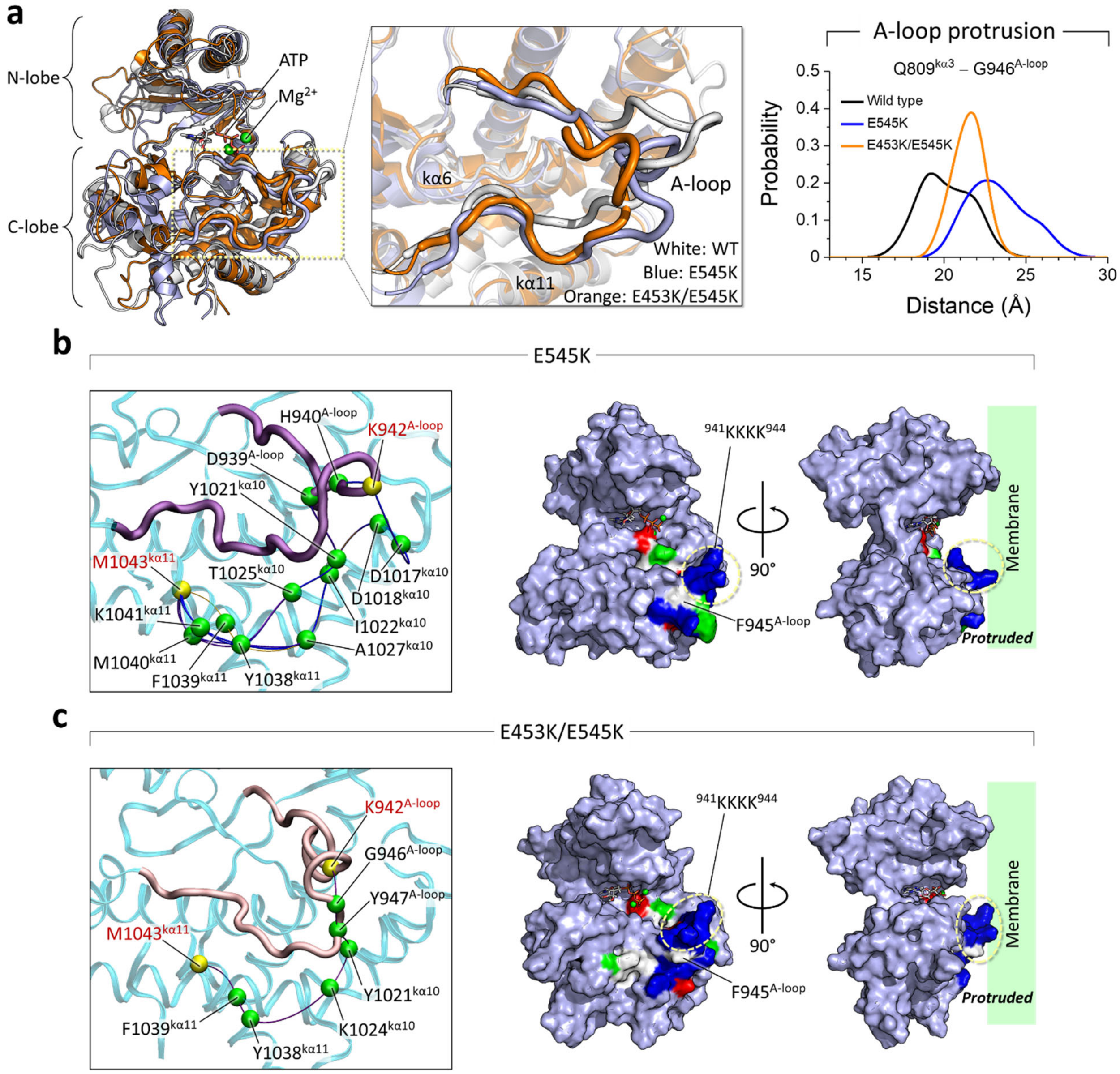

Supplementary Fig. 7 | Both E545K and E453K/E545K mutations also cause the A-loop to protrude.

a, Superimposition of kinase domains from the best representative conformations highlights A-loop conformations. Wild-type is shown in white, the single mutation E545K is shown in blue, and the double mutation E453K/E545K is shown in orange (*left and middle*). The probability distribution of the distance between Q809$^{k\alpha3}$ and G946$^{A\text{-loop}}$ representing the A-loop protrusion (*right*). **b**, The allosteric signaling pathway between the source residue M1043$^{k\alpha11}$ and the sink residue K942$^{A\text{-loop}}$, and the kinase domain structure highlighting the A-loop for PI3Kα^{E545K}. **c**, The same for PI3Kα$^{E453K/E545K}$. In the pathways, yellow beads represent the source and sink residues, while green beads denote the allosteric signal nodes. In the A-loop structure, residues are colored white, green, blue, and red to indicate their hydrophobic, polar, positively charged, and negatively charged properties, respectively. The protruding region of the A-loop toward the pseudo-membrane is marked.

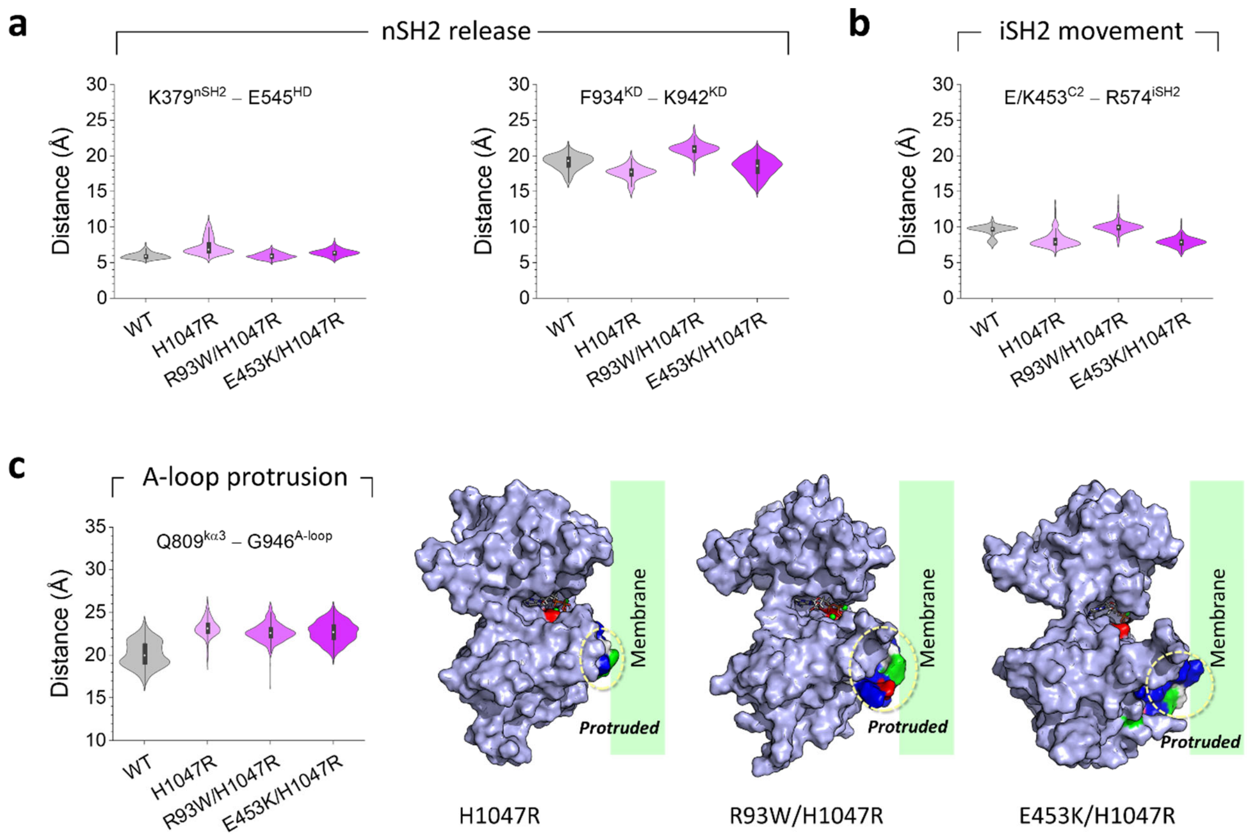

Supplementary Fig. 8 | The H1047R mutation slightly affects the A-loop dynamics.
a, Violin plots representing the atomic pair distances of K379^{nSH2}–E545^{HD} and F934^{KD}–K942^{KD} for PI3Kα^{WT}, PI3Kα^{H1047R}, PI3Kα$^{R93W/H1047R}$, and PI3Kα$^{E453K/H1047R}$, indicating the nSH2 release. HD and KD denote the helical and kinase domains, respectively. **b**, Violin plot representing the atomic pair distance of E/K453^{C2}–R574^{iSH2}, indicating the iSH2 movement. **c**, Violin plot representing the atomic pair distance of Q809$^{k\alpha3}$–G946$^{A\text{-}loop}$, indicating the A-loop protrusion, and the kinase domain structures highlighting the A-loop for the mutations. The kinase domain is colored cyan. In the A-loop structure, residues are colored white, green, blue, and red to indicate their hydrophobic, polar, positively charged, and negatively charged properties, respectively. The protruding region of the A-loop toward the pseudo-membrane is marked.

.

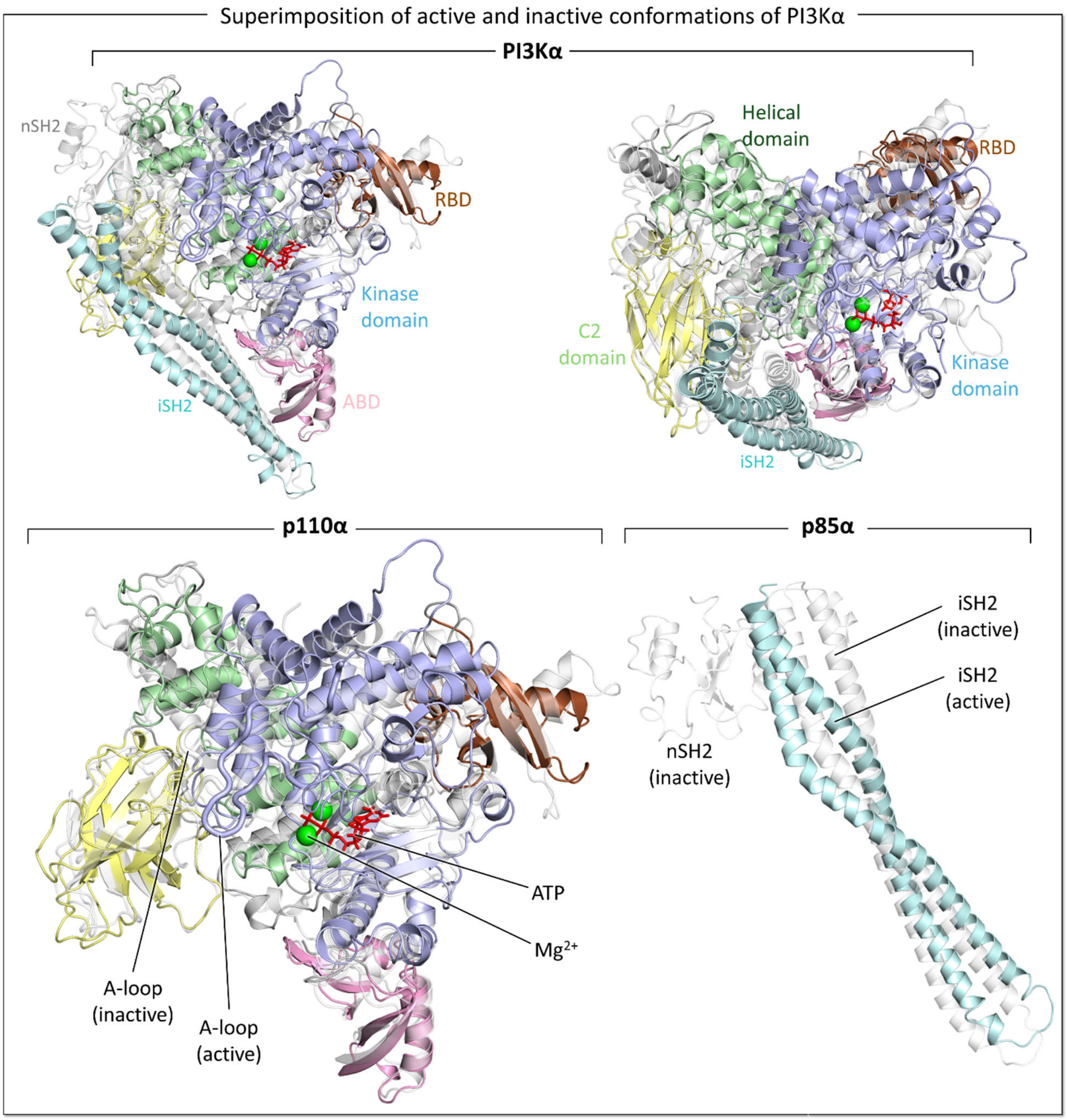

Supplementary Fig. 9 | Active and inactive conformations of PI3Kα differ.
Superimposition of the active and inactive conformations of PI3Kα^{WT}. Blow, the superimposition of the p110α and p85α subunits is highlighted separately. The colored cartoon represents the active form, and the while white, transparent cartoon represents the inactive form.

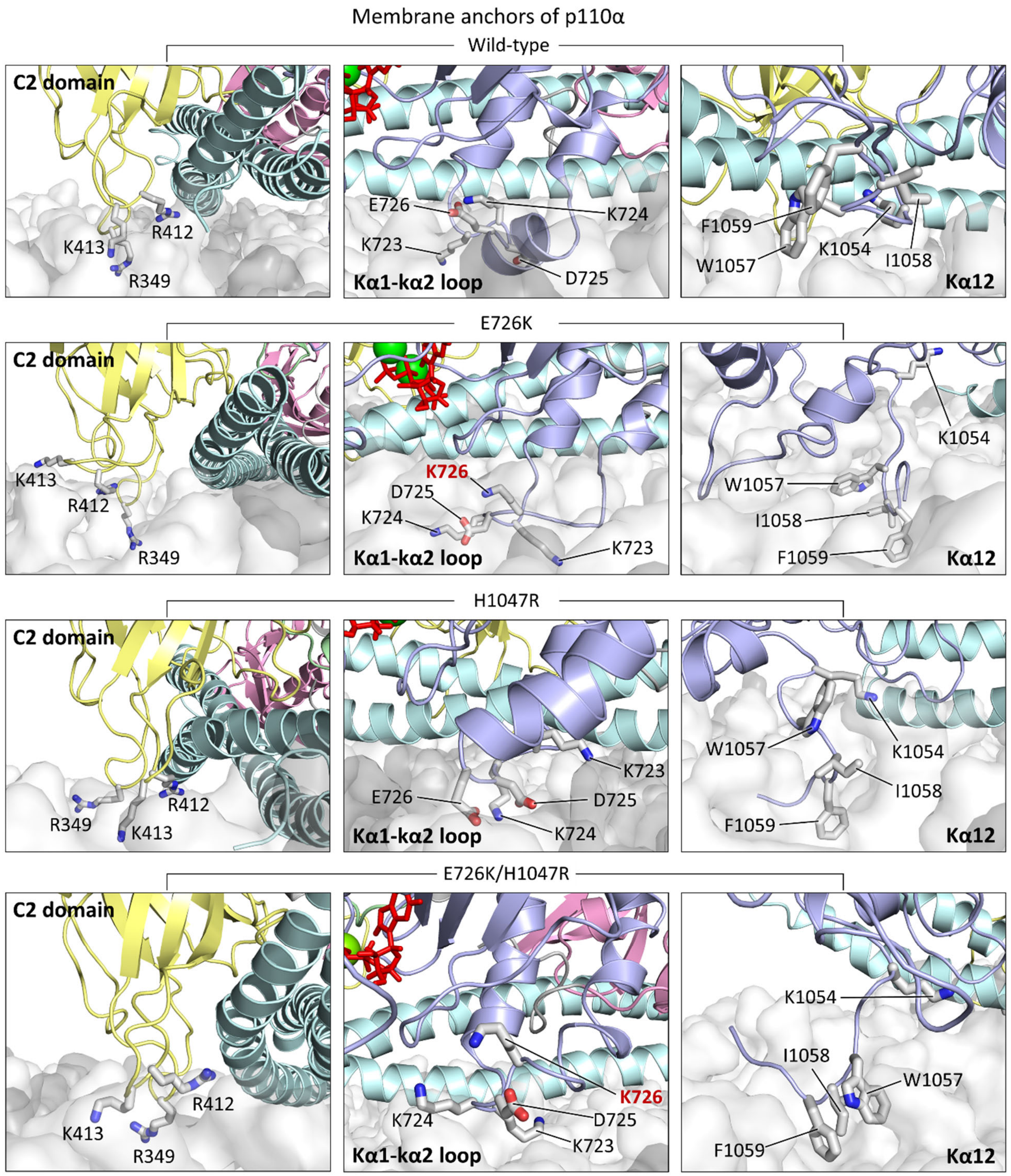

Supplementary Fig. 10 | Membrane interactions of the three anchor points for active PI3Kα. The best representative conformations from the ensemble clusters for PI3Kα^{WT}, PI3Kα^{E726K}, PI3Kα^{H1047R}, and PI3Kα$^{E726K/H1047R}$ are shown, with highlights of the residues interacting with the membrane at the three anchor points located in the C2 domain, the kα1-kα2 loop, and the kα12 helix.

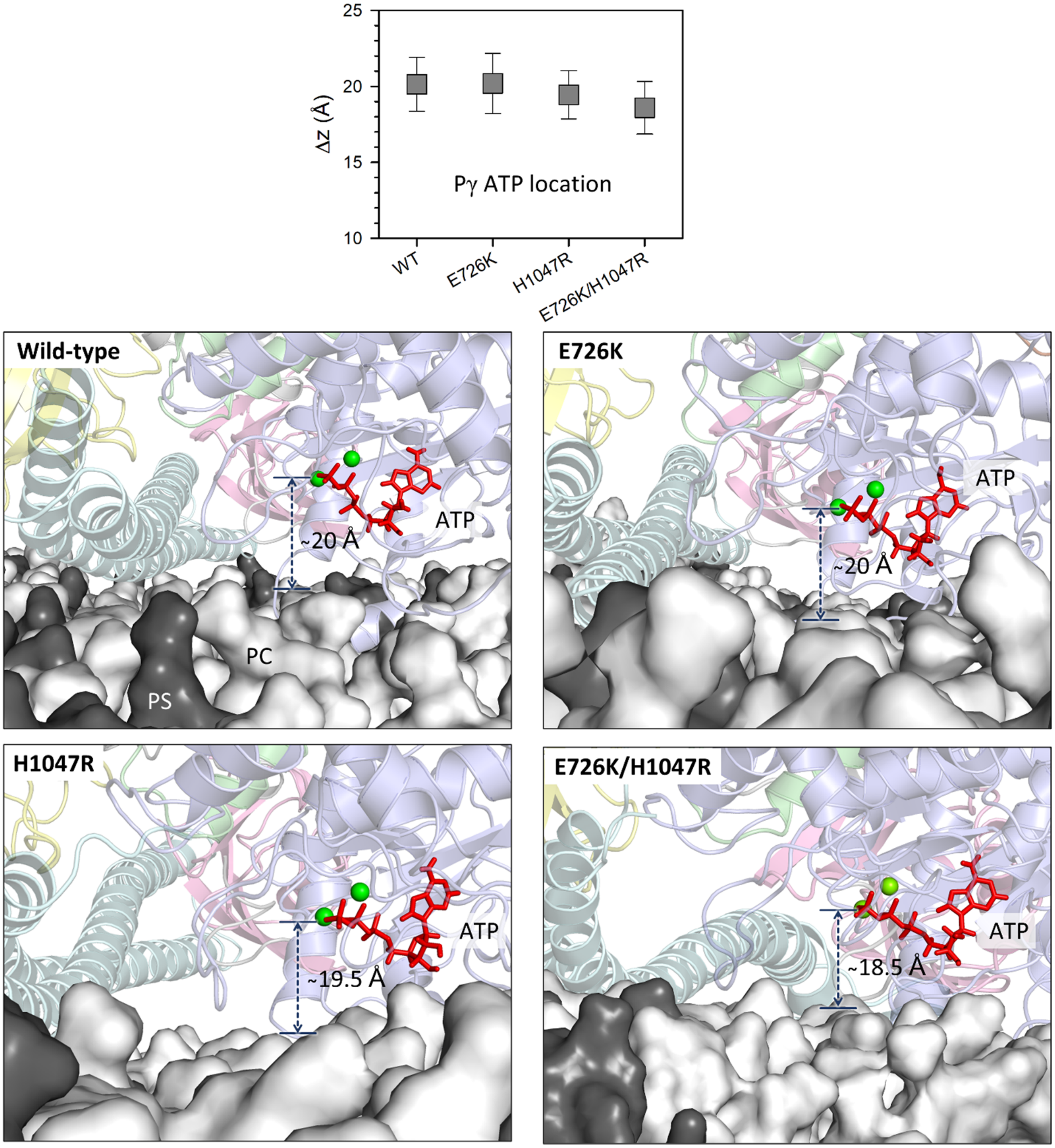

Supplementary Fig. 11 | ATP location from the bilayer surface.

Deviation of Pγ of ATP from the averaged position of the phosphate atoms of DOPC and DOPS for $PI3K\alpha^{WT}$, $PI3K\alpha^{E726K}$, $PI3K\alpha^{H1047R}$, and $PI3K\alpha^{E726K/H1047R}$. Snapshots highlighting ATP with deviations of Pγ of ATP marked on each system.

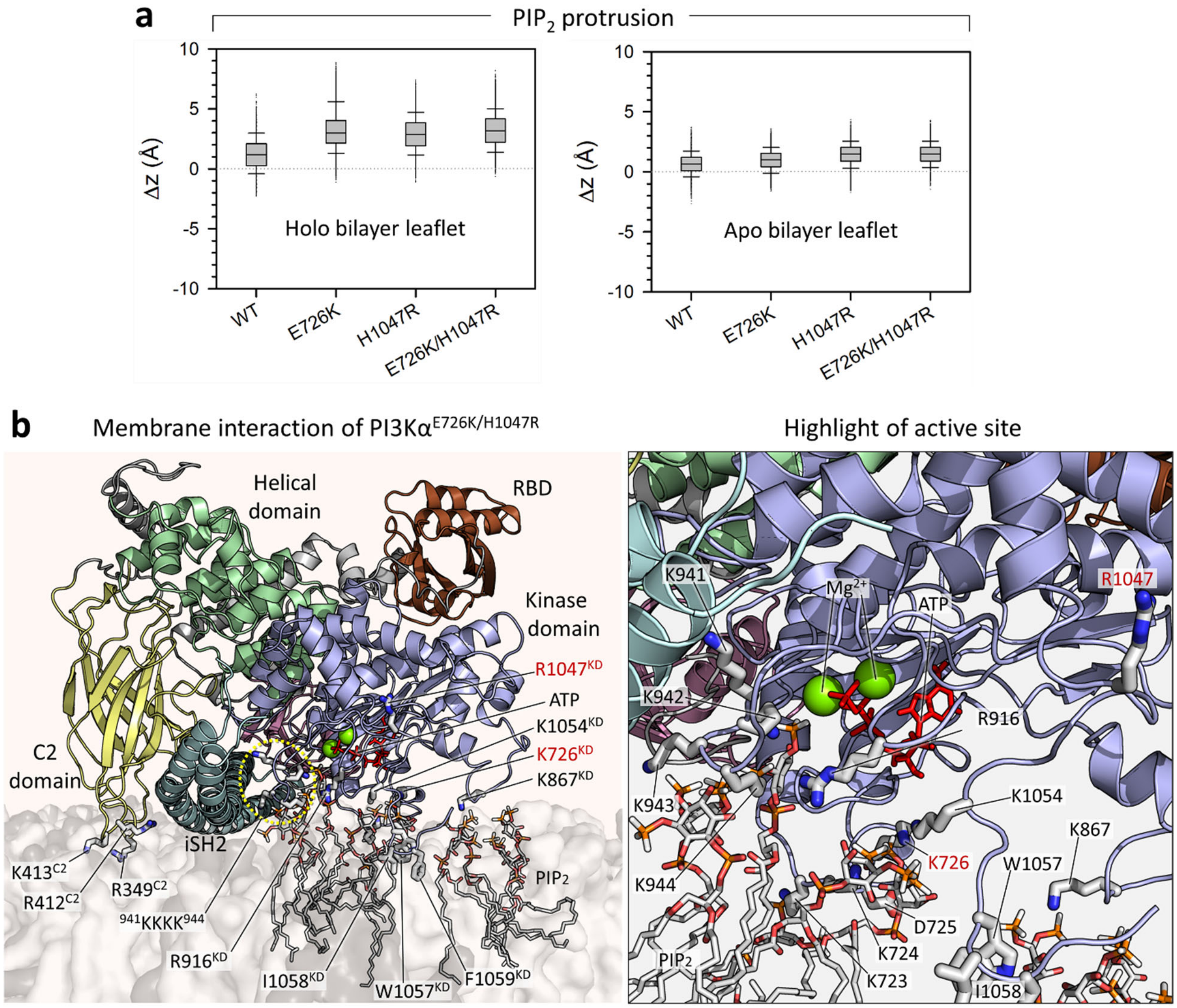

Supplementary Fig. 12 | PIP₂ protrusion.

a, Deviations of the phosphate atoms of PIP_2 from the averaged position of the phosphate atoms of DOPC and DOPS for the *holo* and *apo* bilayer leaflets for $PI3K\alpha^{WT}$, $PI3K\alpha^{E726K}$, $PI3K\alpha^{H1047R}$, and $PI3K\alpha^{E726K/H1047R}$. **b**, Snapshots showing membrane interaction of $PI3K\alpha^{E726K/H1047R}$ and highlighting the active site in the kinase domain. PIP_2 is protruding from the bilayer surface toward the active site. The mutant residues K726 and R1047 are marked in red. The protein structure is depicted as a cartoon with different color codes: The ABD is pink, the RBD is brown, the C2 domain is yellow, the helical domain is green, the kinase domain is blue, and iSH2 is teal. PIP_2 is shown as sticks. The white and gray transparent surfaces represent DOPC and DOPS, respectively. ATP is shown as red sticks, and Mg^{2+} is shown as a green sphere.

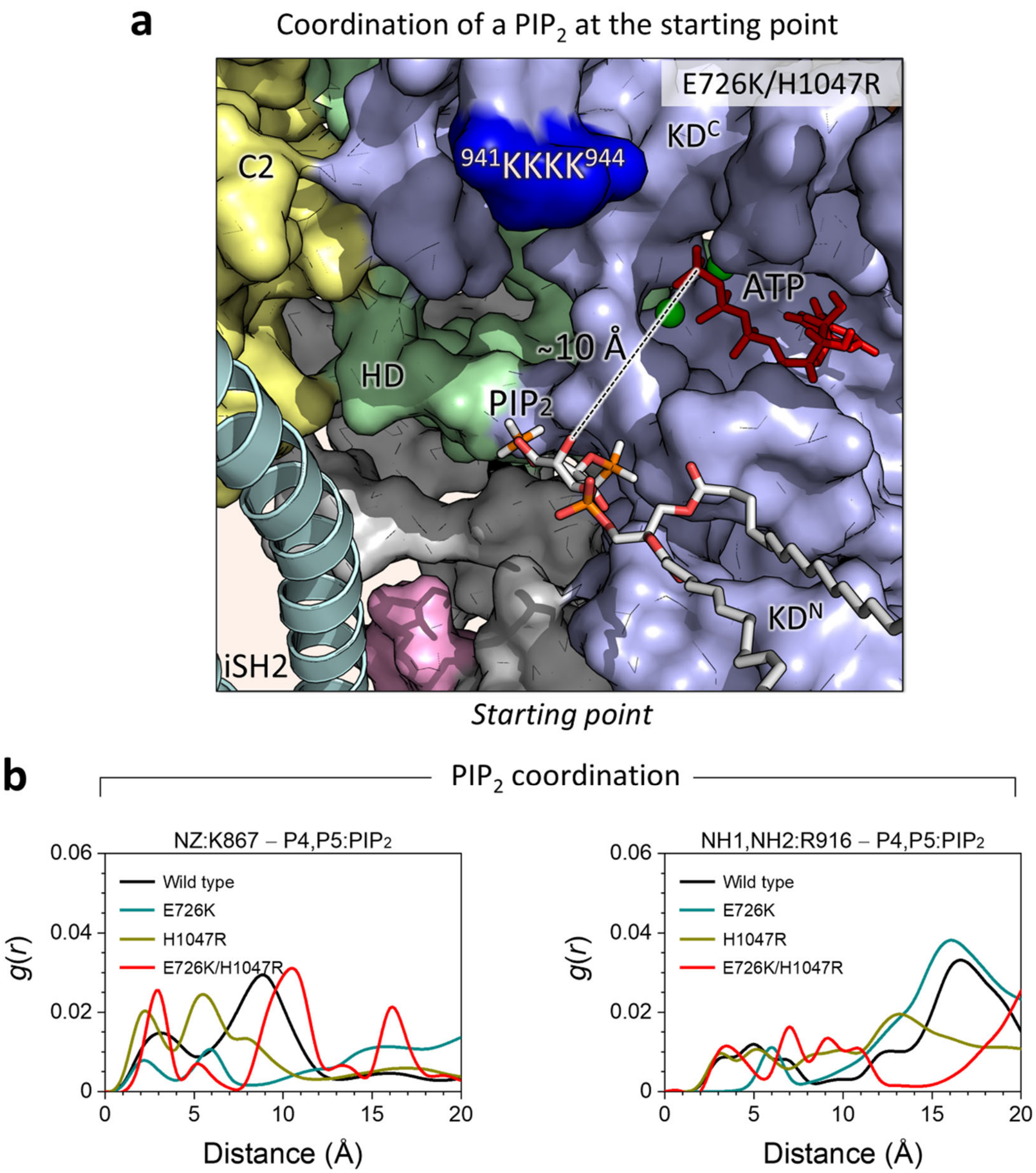

Supplementary Fig. 13 | PIP_2 coordination.
a, Snapshot highlighting the active site of PI3Kα$^{E726K/H1047R}$ at the starting point. A particular PIP_2 is initially placed at the active site with a coordination distance of ~10 Å. The coordination distance is defined as the distance between the hydroxyl group at the 3rd position of the inositol ring of PIP_2 and the Pγ of ATP. The p110α structure is shown in surface representation with different color codes: The C2 domain is yellow, the helical domain is green, and the kinase domain is blue. The p85α structure is depicted as a cartoon. PIP_2 is shown as sticks. ATP is shown as red sticks, and Mg^{2+} is shown as a green sphere. C2 and HD denote the C2 and helical domains, respectively. KD^N and KD^C represent the N-lobe and C-lobe kinase domain, respectively. The dark blue surface highlights the ^{941}KKKK^{944} motif in the A-loop. **b**, The radial distribution function, $g(r)$, of selected atom pairs for PI3Kα^{WT}, PI3Kα^{E726K}, PI3Kα^{H1047R}, and PI3Kα$^{E726K/H1047R}$.

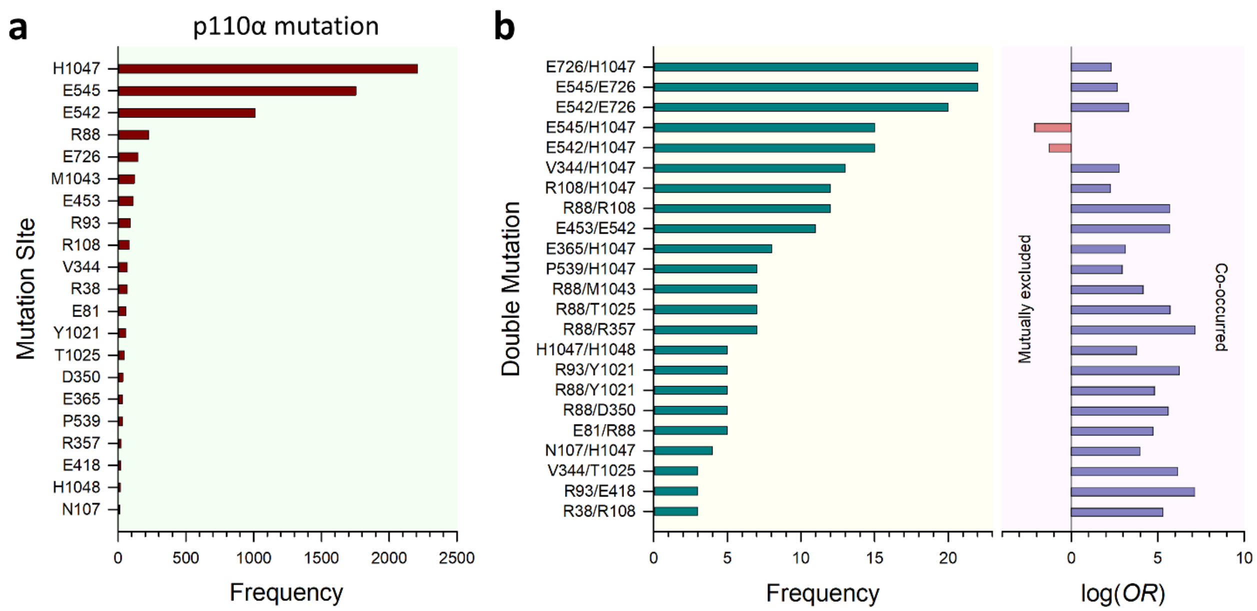

Supplementary Fig. 14 | *PIK3CA* mutations and their frequencies.
a, Statistical analyses based on the frequencies of single mutations in p110α. **b**, Statistical analyses based on the frequencies of double mutations. The red bars on the left indicate mutually exclusive doublets for E545/H1047 and E542/H1047.

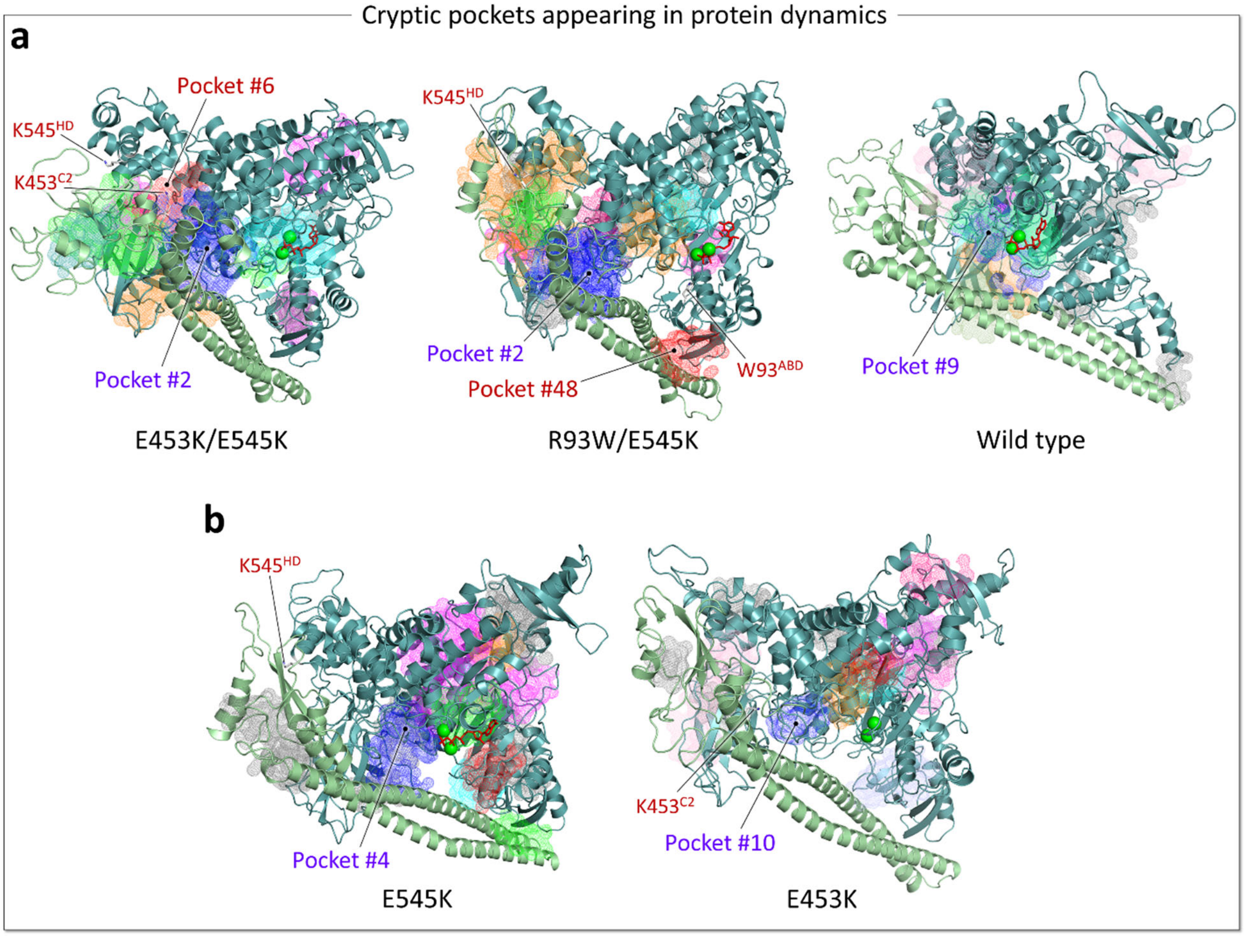

Supplementary Fig. 15 | Discovery of cryptic allosteric pockets in PI3Kα

a, Predicted cryptic allosteric pockets for PI3Kα$^{E453K/E545K}$, PI3Kα$^{R93W/E545K}$, and PI3Kα^{WT}. **b**, Predicted cryptic allosteric pockets for PI3Kα^{E545K} and PI3Kα^{E453K}. The blue mesh represents the pocket with the highest probability, and the red mesh represents the next highest probability pocket. Other possible pocket locations are shown in different colors on a mesh background.

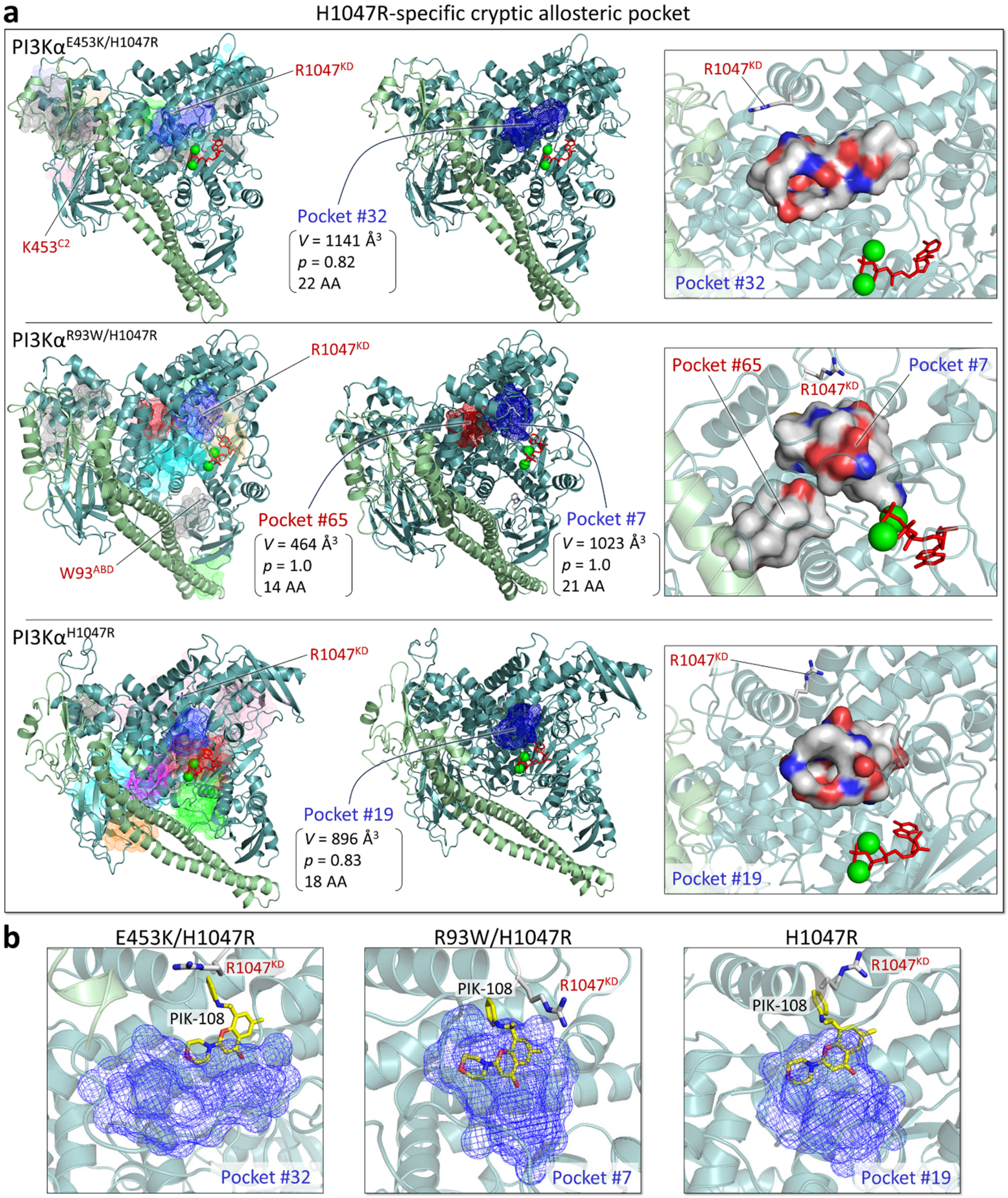

Supplementary Fig. 16 | H1047R-specific cryptic allosteric pockets.

a, Predicted cryptic allosteric pockets for PI3Kα$^{E453K/H1047R}$, PI3Kα$^{R93W/H1047R}$, and PI3Kα^{H1047R} (*left*). The blue mesh represents the pocket with the highest probability, and the red mesh represents the next highest probability pocket. The pocket volume, probability, and number of amino acids involved in the pocket are indicated (*middle*). Highlights of these pockets are shown in the surface

representation (*right*). In the pocket structure, residues are colored white, green, blue, and red to indicate their hydrophobic, polar, positively charged, and negatively charged properties, respectively. **b**, Overlays of the allosteric drug PIK-108 in the pockets predicted for the E453K/H1047R (*left*), R93W/H1047R (*middle*), and H1047R (*right*) mutations.

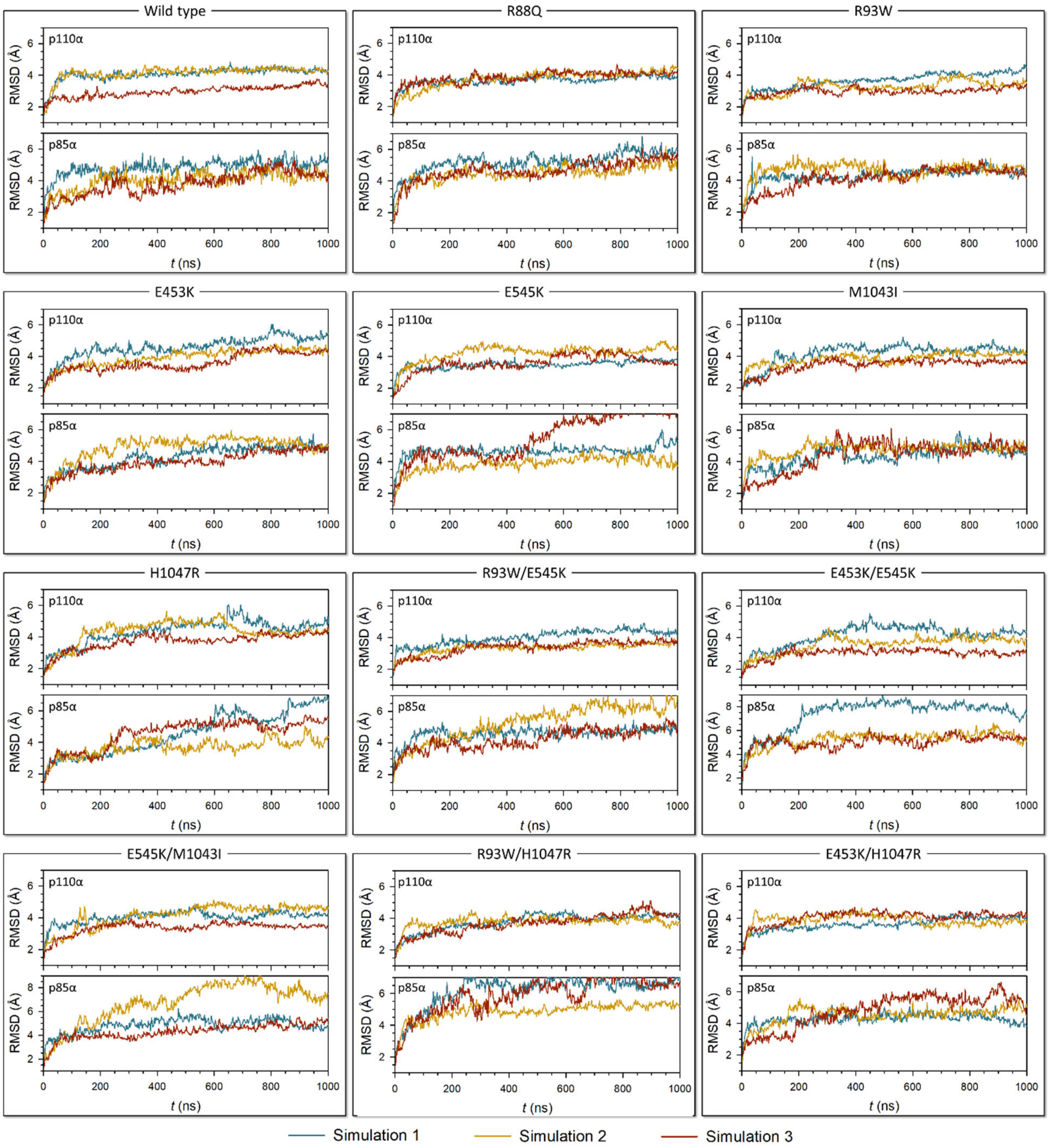

Supplementary Fig. 17 | RMSD of p110α and p85α subunits.
Time series showing the root-mean-squared-deviations (RMSDs) of the p110α and p85α subunits for three replica simulations of each system in solution.

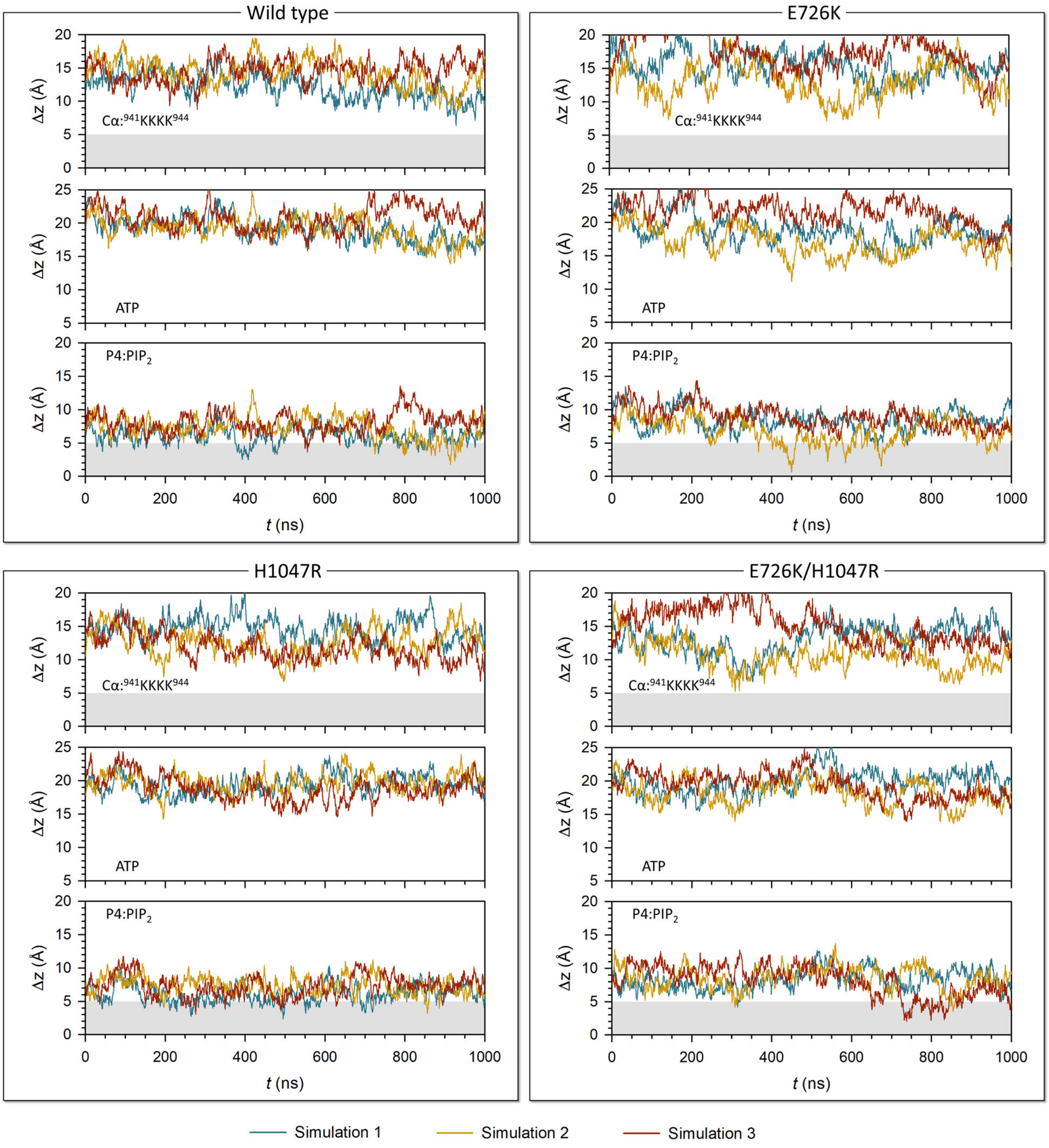

Supplementary Fig. 18 | Location of the $_{941}$KKKK$_{944}$ motif in the A-loop, ATP, and PIP_2 at the membrane.

Time series showing the deviations of the Cα atoms in the $_{941}$KKKK$_{944}$ motif in the A-loop, the center of mass of ATP, and the phosphate at the 4th position of the inositol ring of PIP_2 from the bilayer surface for three replica simulations of each system.

Supplementary Table 1. *PIK3CA* mutations and their functions, and diseases associated with them. Oncogenic hotspot mutations are in red.

p110α mutation Interpretation (germline) [somatic]	CDS mutation Genomic location (GRCh38) [GRCh37]	Function	Disease	Reference (PMID)
**R88Q**<br><br>(Pathogenic)<br>[Oncogenic]	c.263G>A<br><br>(3: 179199088)<br>[3: 178916876]	Gain of function mutation, increased AKT phosphorylation, downstream target rpS6, cell proliferation, migration, and transformation ability	Ovarian neoplasm, Cowden syndrome, megalencephaly-capillary malformation-polymicrogyria syndrome, PROS, abnormal cerebral morphology, CLOVES syndrome, neoplasm	18829572, 22729224 22949682, 28941273, 29296277, 29533785, 32778138, 34779417, 39825153
**R93W**<br><br>(Pathogenic/Likely pathogenic)<br>[Likely oncogenic]	c.277C>T<br><br>(3: 179199102)<br>[3: 178916890]	Gain of function mutation, increased AKT phosphorylation and transformation ability	Megalencephaly-capillary malformation-polymicrogyria syndrome, PROS, neoplasm	21266528, 27191687, 27631024, 28191889, 29533785, 32733937, 34733958
**E453K**<br><br>(Pathogenic)<br>[No somatic data]	c.1357G>A<br><br>(3: 179210291)<br>[3: 178928079]	Gain of function mutation, increased AKT and MEK1/2 phosphorylation, cell proliferation, migration, and transformation ability, and enhanced cell survival	Ovarian neoplasm, Cowden syndrome, megalencephaly-capillary malformation-polymicrogyria syndrome, CLOVES syndrome, abnormal cardiovascular system morphology, PROS	22729224, 26627007, 27631024, 28151489, 29533785, 30063105, 34779417, 36474027
**E545K**<br><br>(Pathogenic/Likely pathogenic)<br>[Oncogenic]	c.1633G>A<br><br>(3: 179218303)<br>[3: 178936091]	Gain of function mutation, increased AKT and MEK1/2 phosphorylation, growth factor-independent cell survival, and transformation ability	Epithelial ovarian cancer, breast adenocarcinoma, carcinoma of colon, seborrheic keratosis, ovarian neoplasm, non-small cell lung carcinoma, megalencephaly-capillary malformation-polymicrogyria syndrome, sarcoma, segmental undergrowth, gallbladder cancer, neoplasm	23946963,25880439, 26627007, 27631024, 28151489, 28425981, 29533785, 29661094, 30376034, 30543347, 31536475, 37712948
**E726K**<br><br>(Pathogenic)<br>[No somatic data]	c.2176G>A<br><br>(3: 179221146)<br>[3: 178938934]	Gain of function mutation, increased cell proliferation, migration, AKT phosphorylation, and transformation ability	Inborn genetic diseases, overgrowth syndrome and/or cerebral malformations, Cowden syndrome, megalencephaly-capillary malformation-polymicrogyria syndrome, PROS	22729224, 23246288, 24497998, 26351730, 27631024, 28151489, 28566443, 28941273, 29533785, 29549527, 34779417
**M1043I**<br><br>(Pathogenic)<br>[No somatic data]	c.3129G>T<br><br>(3: 179234286)<br>[3: 178952074]	Gain of function mutation, increased AKT phosphorylation, activation of downstream signaling, and transformation	Cowden syndrome, PROS,	15930273, 17376864, 22120714, 22729224, 29533785
**H1047R**<br><br>(Pathogenic)<br>[Oncogenic]	c.3140A>G<br><br>(3: 179234297)<br>[3: 178952085]	Gain of function mutation, increased AKT and MEK1/2 phosphorylation, growth factor-independent cell survival, and transformation ability	Epithelial ovarian cancer, breast adenocarcinoma, carcinoma of colon, hepatocellular carcinoma, non-small cell lung carcinoma, seborrheic keratosis, CLOVES syndrome, macrodactyly, ovarian neoplasm, neoplasm	23946963, 26266985, 26627007, 27126994, 29533785, 29988677, 31371346, 31536475, 34008892, 34496175, 39825153

*Data were imported from ClinVar (https://www.ncbi.nlm.nih.gov/clinvar) and the Jackson Laboratory Clinical Knowledgebase (CKB, https://ckb.jax.org/gene/grid).

Supplementary Table 2. Properties of the predicted cryptic pockets for PI3Kα$^{E453K/E545K}$. Here, P_{Hydro}, P_{Polar}, P_{Aroma}, and P_{Alipha} correspond to the proportions of hydrophobic, polar, aromatic, and aliphatic residues in the pockets, respectively. GRAVY denotes the grand average of hydropathy (doi: 10.1016/0022-2836(82)90515-0). This is related to Fig. 8a and Supplementary Fig. 15a.

Pocket	P_{Hydro}	P_{Polar}	P_{Aroma}	P_{Alipha}	Volume (Å^3)	Surface (Å^2)	Diameter (Å)	GRAVY	N_{Res}	*p*
**#2**	**0.59**	**0.62**	**0.18**	**0.18**	**11603**	**2936**	**54**	**-0.90**	**71**	**0.75**
**#6**	**0.55**	**0.52**	**0.15**	**0.23**	**3710**	**1157**	**24**	**-0.58**	**40**	**0.63**
#38	0.88	0.25	0.31	0.44	632	418	18	1.83	16	0.99
#50	0.80	0.13	0.13	0.60	575	364	14	2.19	15	0.99
#35	0.71	0.43	0.29	0.33	1082	574	20	0.74	21	0.98
#4	0.71	0.38	0.15	0.35	2645	1070	25	0.91	34	0.98
#13	0.63	0.51	0.14	0.29	2221	967	29	0.15	35	0.90
#21	0.71	0.52	0.23	0.23	2806	1091	26	-0.18	31	0.86
#53	0.73	0.64	0.36	0.14	1839	689	21	-0.64	22	0.82
#1	0.65	0.54	0.11	0.28	5900	1788	32	-0.27	57	0.80
#23	0.65	0.41	0.06	0.24	709	425	15	0.18	17	0.78
#7	0.64	0.61	0.21	0.14	2540	999	24	-0.82	28	0.53

Supplementary Table 3. Properties of the predicted cryptic pockets for PI3Kα$^{R93W/E545K}$. See Supplementary Table 2 for descriptions of the parameters used in the table. This is related to Fig. 8b and Supplementary Fig. 15a.

Pocket	P_{Hydro}	P_{Polar}	P_{Aroma}	P_{Alipha}	Volume (Å^3)	Surface (Å^2)	Diameter (Å)	GRAVY	N_{Res}	*p*
**#2**	**0.61**	**0.58**	**0.18**	**0.17**	**9043**	**2359**	**39**	**-0.92**	**71**	**0.65**
**#48**	**0.58**	**0.58**	**0.21**	**0.21**	**1638**	**811**	**25**	**-0.35**	**19**	**0.75**
#12	0.69	0.38	0.19	0.44	805	463	17	1.58	16	0.99
#39	0.81	0.25	0.31	0.50	713	441	18	1.85	16	0.99
#65	0.88	0.13	0.19	0.56	623	394	14	2.5	16	0.99
#46	0.80	0.40	0.27	0.27	607	389	16	0.63	15	0.98
#42	0.79	0.50	0.36	0.21	751	479	18	-0.21	14	0.88
#53	0.50	0.50	0	0.36	459	339	16	0.59	14	0.88
#7	0.59	0.47	0.24	0.24	855	477	17	0.08	17	0.87
#18	0.76	0.53	0.24	0.24	770	457	16	0.04	17	0.86
#29	0.60	0.53	0.10	0.23	2432	974	25	-0.14	30	0.78
#49	0.57	0.50	0.29	0.21	666	412	17	-0.43	14	0.77
#1	0.59	0.60	0.12	0.21	13630	3078	42	-0.68	91	0.74
#9	0.57	0.57	0.07	0.29	510	335	14	-0.36	14	0.50

Supplementary Table 4. Properties of the predicted cryptic pockets for PI3Kα^{E545K}. See Supplementary Table 2 for descriptions of the parameters used in the table. This is related to Supplementary Fig. 15b.

Pocket	P_{Hydro}	P_{Polar}	P_{Aroma}	P_{Alipha}	Volume (Å^3)	Surface (Å^2)	Diameter (Å)	GRAVY	N_{Res}	*p*
**#4**	**0.63**	**0.60**	**0.23**	**0.15**	**3862**	**1297**	**25**	**-0.88**	**40**	**0.60**
#62	0.86	0.14	0.21	0.50	435	312	15	2.39	14	0.99
#64	0.78	0.39	0.11	0.39	1043	591	22	0.98	18	0.98
#9	0.67	0.48	0.14	0.38	1184	604	20	0.73	21	0.97
#12	0.68	0.39	0.18	0.36	1941	855	24	0.50	28	0.95
#27	0.75	0.56	0.38	0.13	658	419	18	-0.23	16	0.93
#41	0.67	0.47	0.27	0.33	688	421	15	-0.01	15	0.90
#20	0.61	0.56	0.17	0.22	688	422	16	-0.16	18	0.80
#51	0.71	0.57	0.29	0.21	642	428	19	-0.39	14	0.80
#22	0.63	0.63	0.25	0.25	805	466	16	-0.23	16	0.78
#32	0.71	0.47	0.06	0.35	918	521	19	0.11	17	0.78
#8	0.58	0.58	0.17	0.25	1935	824	21	-0.29	24	0.72
#6	0.62	0.62	0.17	0.24	5280	1709	32	-0.64	42	0.67
#16	0.53	0.59	0.12	0.18	741	428	15	-0.37	17	0.56

Supplementary Table 5. Properties of the predicted cryptic pockets for PI3Kα^{E453K}. See Supplementary Table 2 for descriptions of the parameters used in the table. This is related to Supplementary Fig. 15b.

Pocket	P_{Hydro}	P_{Polar}	P_{Aroma}	P_{Alipha}	Volume (Å^3)	Surface (Å^2)	Diameter (Å)	GRAVY	N_{Res}	*p*
**#10**	**0.69**	**0.56**	**0.19**	**0.19**	**876**	**494**	**17**	**-0.31**	**16**	**0.69**
#45	0.86	0.29	0.21	0.36	480	332	14	2.16	14	0.99
#52	0.80	0.27	0.20	0.53	680	417	15	2.01	15	0.99
#27	0.93	0.40	0.33	0.27	574	379	15	0.71	15	0.98
#36	0.80	0.33	0	0.40	784	466	16	0.99	15	0.95
#24	0.64	0.50	0.21	0.36	365	271	11	0.34	14	0.91
#63	0.58	0.47	0.21	0.26	1123	595	18	-0.19	19	0.84
#3	0.63	0.57	0.14	0.29	5653	1810	37	-0.32	49	0.82
#7	0.64	0.64	0.29	0.21	879	516	18	-0.30	14	0.78
#33	0.64	0.57	0.21	0.21	544	359	14	-0.35	14	0.74
#6	0.61	0.67	0.17	0.17	920	523	17	-0.36	18	0.70
#54	0.57	0.50	0.07	0.36	598	384	15	-0.14	14	0.68
#12	0.61	0.56	0.28	0.17	910	504	17	-0.52	18	0.67
#41	0.53	0.47	0.12	0.29	1042	569	19	-0.32	17	0.63

Supplementary Table 6. Properties of the predicted cryptic pockets for PI3Kα^{WT}. See Supplementary Table 2 for descriptions of the parameters used in the table. This is related to Fig. 8c and Supplementary Fig. 15a.

Pocket	P_{Hydro}	P_{Polar}	P_{Aroma}	P_{Alipha}	Volume (Å^3)	Surface (Å^2)	Diameter (Å)	GRAVY	N_{Res}	*p*
**#9**	**0.71**	**0.60**	**0.20**	**0.17**	**3475**	**1266**	**29**	**-0.81**	**35**	**0.58**
#42	0.87	0.20	0.20	0.67	576	366	13	2.5	15	0.99
#17	0.60	0.43	0.20	0.33	2040	891	23	0.67	30	0.97
#21	0.72	0.48	0.68	0.24	1860	846	25	0.68	25	0.96
#16	0.68	0.58	0.26	0.26	2798	1097	25	-0.09	31	0.91
#11	0.72	0.50	0.19	0.25	3824	1337	29	-0.08	36	0.91
#2	0.69	0.51	0.15	0.26	6509	1912	36	-0.10	61	0.90
#14	0.70	0.48	0.15	0.27	2391	978	25	0.08	33	0.87
#7	0.69	0.64	0.23	0.15	4050	1402	32	-0.38	39	0.86
#35	0.64	0.50	0.14	0.36	612	398	15	-0.24	14	0.72
#22	0.63	0.42	0.11	0.11	844	486	17	-0.08	19	0.72
#25	0.53	0.40	0.07	0.40	595	378	14	0.01	15	0.71
#5	0.55	0.68	0.18	0.20	3919	1328	27	-0.96	40	0.51

Supplementary Table 7. Properties of the predicted cryptic pockets for PI3Kα$^{E453K/H1047R}$. See Supplementary Table 2 for descriptions of the parameters used in the table. This is related to Supplementary Fig. 16a.

Pocket	P_{Hydro}	P_{Polar}	P_{Aroma}	P_{Alipha}	Volume (Å^3)	Surface (Å^2)	Diameter (Å)	GRAVY	N_{Res}	*p*
**#32**	**0.73**	**0.50**	**0.23**	**0.27**	**1141**	**618**	**23**	**-0.32**	**22**	**0.82**
#19	0.71	0.29	0.12	0.53	681	414	15	2.03	17	0.99
#79	0.99	0.07	0.29	0.57	629	396	15	3.19	14	0.99
#13	0.75	0.44	0.31	0.31	565	362	14	0.90	16	0.98
#49	0.71	0.43	0.07	0.43	548	372	18	1.13	14	0.98
#12	0.73	0.57	0.30	0.33	2763	1108	28	0.04	30	0.95
#3	0.65	0.41	0.24	0.29	980	522	16	0.52	17	0.95
#8	0.76	0.47	0.18	0.35	609	381	14	0.52	17	0.94
#7	0.71	0.43	0.21	0.29	579	386	14	0	14	0.83
#43	0.59	0.59	0.18	0.18	1502	723	22	-0.40	22	0.74
#76	0.59	0.53	0.18	0.24	853	480	18	-0.22	17	0.73
#26	0.53	0.53	0.12	0.29	669	422	16	-0.37	17	0.57
#18	0.64	0.50	0	0.29	901	507	18	-0.22	14	0.53

Supplementary Table 8. Properties of the predicted cryptic pockets for PI3Kα$^{R93W/H1047R}$. See Supplementary Table 2 for descriptions of the parameters used in the table. This is related to Supplementary Fig. 16a.

Pocket	P_{Hydro}	P_{Polar}	P_{Aroma}	P_{Alipha}	Volume (Å^3)	Surface (Å^2)	Diameter (Å)	GRAVY	N_{Res}	*p*
**#7**	**0.90**	**0.38**	**0.33**	**0.29**	**1023**	**551**	**18**	**1.53**	**21**	**0.99**
**#65**	**0.99**	**0**	**0.21**	**0.57**	**464**	**339**	**14**	**3.46**	**14**	**0.99**
#35	0.87	0.40	0.07	0.47	974	547	18	1.03	15	0.97
#4	0.65	0.45	0.25	0.30	1311	640	20	0.43	20	0.95
#42	0.73	0.53	0.13	0.40	656	420	16	0.65	15	0.94
#32	0.67	0.47	0.13	0.27	587	385	16	0.39	15	0.92
#15	0.65	0.59	0.18	0.18	622	407	17	-0.06	17	0.79
#31	0.71	0.53	0.24	0.24	1168	603	20	-0.34	17	0.78
#16	0.58	0.58	0.16	0.26	1098	579	18	-0.19	19	0.74
#53	0.60	0.60	0.20	0.33	801	497	18	-0.58	15	0.62

Supplementary Table 9. Properties of the predicted cryptic pockets for PI3Kα^{H1047R}. See Supplementary Table 2 for descriptions of the parameters used in the table. This is related to Supplementary Fig. 16a.

Pocket	P_{Hydro}	P_{Polar}	P_{Aroma}	P_{Alipha}	Volume (Å^3)	Surface (Å^2)	Diameter (Å)	GRAVY	N_{Res}	*p*
**#19**	**0.72**	**0.56**	**0.33**	**0.22**	**896**	**503**	**18**	**-0.24**	**18**	**0.83**
#53	0.80	0.40	0.27	0.33	758	470	19	0.57	15	0.97
#6	0.74	0.48	0.39	0.21	1347	650	20	0.07	23	0.97
#7	0.79	0.57	0.21	0.36	591	389	16	0.72	14	0.97
#10	0.68	0.50	0.23	0.27	1015	548	18	0.22	22	0.94
#11	0.69	0.46	0.23	0.31	1697	819	26	0.22	26	0.94
#2	0.59	0.49	0.13	0.28	7442	2116	34	-0.16	61	0.87
#41	0.69	0.50	0.13	0.13	607	393	17	0.09	16	0.81
#25	0.47	0.59	0.18	0.18	763	457	16	-0.38	17	0.63
#3	0.58	0.54	0.10	0.17	5948	1780	34	-0.60	48	0.63
#18	0.51	0.54	0.09	0.23	3869	1349	31	-0.53	35	0.59
#38	0.57	0.57	0.14	0.21	1304	662	20	-0.47	14	0.58

Supplementary Table 10. Details of simulation systems. Oncogenic hotspot mutations are in red.

System	Mutation site in p110α	Mutation type	Simulation
Wild type	–	–	• Started with an inactive PI3Kα conformation in solution<br>• Performed 3 replica simulations, each with 1 μs simulation<br>• TIP3P water in a cubic box
R88Q	ABD	Moderate	
R93W	ABD	Weak	
E453K	C2	Weak	
E545K	Helical	Hotspot	
M1043I	KD (C-term)	Weak	
H1047R	KD (C-term)	Hotspot	
R93W/E545K	ABD/Helical	Weak/Hotspot	
E453K/E545K	C2/Helical	Weak/Hotspot	
M1043I/E545K	KD/Helical	Weak/Hotspot	
R93W/H1047R	ABD/KD	Weak/Hotspot	
E453K/H1047R	C2/KD	Weak/Hotspot	
Wild type	–	–	• Started with an active PI3Kα conformation with truncated nSH2 in the membrane<br>• Performed 3 replica simulations, each with 1 μs simulation<br>• Anionic lipid bilayer composed of DOPC:DOPS:PIP_2 (28:6:1 molar ratio)
E726K	KD (N-term)	Weak	
H1047R	KD (C-term)	Hotspot	
E726K/H1047R	KD/KD	Weak/Hotspot	
